# Abstract withdrawn

**DOI:** 10.1186/1753-6561-6-S4-O12

**Published:** 2012-07-09

## Abstract withdrawn

